# Ablation of CCAAT/Enhancer-Binding Protein Delta (C/EBPD): Increased Plaque Burden in a Murine Alzheimer’s Disease Model

**DOI:** 10.1371/journal.pone.0134228

**Published:** 2015-07-31

**Authors:** Manuel Lutzenberger, Michael Burwinkel, Constanze Riemer, Victoria Bode, Michael Baier

**Affiliations:** Research Group Proteinopathies/Neurodegenerative Diseases, Centre for Biological Threats and Special Pathogens (ZBS6), Robert Koch-Institut, Berlin, Germany; University of S. Florida College of Medicine, UNITED STATES

## Abstract

Alzheimer’s disease (AD) and prion diseases carry a significant inflammatory component. The astrocytic overexpression of CCAAT/enhancer-binding protein delta (C/EBPD) in prion- and AD-affected brain tissue prompted us to study the role of this transcription factor in murine model systems of these diseases. Ablation of C/EBPD had neither in the AD model (APP/PS1double transgenic mice) nor in the prion model (scrapie-infected C57BL/6 mice) an influence on overt clinical symptoms. Moreover, the absence of C/EBPD did not affect the extent of the disease-related gliosis. However, C/EBPD-deficient APP/PS1 double transgenic mice displayed significantly increased amyloid beta (Abeta) plaque burdens while amyloid precursor protein (APP) expression and expression of genes involved in beta amyloid transport and turnover remained unchanged. Gene expression analysis in mixed glia cultures demonstrated a strong dependency of complement component C3 on the presence of C/EBPD. Accordingly, C3 mRNA levels were significantly lower in brain tissue of C/EBPD-deficient mice. Vice versa, C3 expression in U-373 MG cells increased upon transfection with a C/EBPD expression vector. Taken together, our data indicate that a C/EBPD-deficiency leads to increased Abeta plaque burden in AD model mice. Furthermore, as shown in vivo and in vitro, C/EBPD is an important driver of the expression of acute phase response genes like C3 in the amyloid-affected CNS.

## Introduction

CCAAT/enhancer-binding protein delta (C/EBPD) is a member of the CCAAT/enhancer-binding protein (C/EBP) family of transcription factors, which carry a conserved basic-leucine zipper domain directing dimerization and DNA binding. It is involved in the regulation of a diverse range of biological processes like inflammation and cellular differentiation. Basal C/EBPD expression levels are typically low but are highly inducible by multiple stimuli [1]. In the periphery its role in the regulation of acute phase genes like complement component C3 and antichymotrypsin and, more generally, in innate immunity has been extensively studied. E.g. LPS stimulation of TLR-4 on macrophages rapidly induces C/EBPD transcription, which in turn is critical for achieving maximal transcription levels of numerous other genes including IL-6 [2]. Furthermore, C/EBPD is inducible by cytokines like IL-1beta and TNFalpha [3]. Conversely, C/EBPD promotes expression of these pro-inflammatory cytokines thereby potentially forming an autocrine pro-inflammatory feedback mechanism [1]. Given that C/EBPD acts in a self-promoting manner it could potentially contribute to the undesirable prolongation of transient innate immune responses. Activating transcription factor 3 (ATF-3) was identified as transcriptional repressor to limit such C/EBPD-driven responses in the periphery once the inducing bacterial pathogen has been cleared [2].

In the healthy brain C/EBPD was suggested to play a role in the consolidation of long-term memory and to regulate the expression of glycogen metabolism-related enzymes in astrocytes [4, 5]. In the diseased brain C/EBPD was implicated in the progression of neuroinflammation [6–9]. In fact, astrocytic C/EBPD is inducible by pro-inflammatory cytokines like IL-1 [10, 11] and TNFalpha [12]. Accordingly, astrocytic C/EBPD overexpression was observed in brain tissue obtained from AD patients [7], as well as in a murine AD-model [10], and scrapie-infected mice [13]. Moreover, the pronounced induction of C/EBPD expression following traumatic brain injury (TBI) was suggested as link between TBI and the increased risk for subsequent development of AD [14]. In fact, potential target genes of C/EBPD like C3 are overexpressed in AD and have been implicated as disease modifiers.

To gain more insights into functions of C/EBPD in chronic neurodegeneration we investigated here the effects of a C/EBPD deficiency in murine AD and prion disease models and in cell cultures.

## Materials and Methods

### Animals

All animal experiments were approved by the local animal welfare authority (Landesamt für Gesundheit und Soziales, Berlin, Germany). C/EBPD-deficient (C/EBPD^(-/-)^) C57BL/6 mice [15] were obtained from E. Sterneck (National Cancer Institute, Frederick, USA) and outbred to yield C/EBPD^(-/-)^ and C/EBPD^(+/+)^ mice. Transgenic APP/PS1APP_Swe_/PS1dE9 (APP/PS1) mice (background C57BL/6) [16], carrying the human amyloid precursor protein (APP) 695 with the Swedish double mutation K595N/M596L and the human PS1 gene with the exon 9 deletion mutation were from The Jackson Laboratory (stock no. 004462). APP/PS1 mice deficient for C/EBPD (APP/PS1 x C/EBPD^(-/-)^) were generated by crossbreeding APP/PS1 mice with C/EBPD^(-/-)^ mice. Genotyping of C/EBPD^(-/-)^ mice was performed by PCR using the following primers: Wild type (C/EBPD^(+/+)^) mice: CTCCAGGCTTGGACGGCTAAGTAGG (forward) and AAGTTGGCTGTCACCTCGCC (reverse) to detect a 205 bp fragment of the C/EBPD coding region; C/EBPD(-/-) mice: GCTCCAGACTGCCTGGGAAAAGC (forward) and CAGTCCAGTGCCCAAGCTGC (reverse) to amplify a 305 bp fragment of the pGKneo promoter and 3’UTR of C/EBPD. The primer sequences for genotyping the APP/PS1 mice were: GACTGACCACTCGACCAGGTTCTG (forward) and CTTGTAAGTTGGATTCTCATATCCG (reverse) to yield a 350 bp fragment.

The health status of AD mice was checked regularly including determination of body weights, and nest building was assessed as described elsewhere [17].

Scrapie infections were performed as previously described [18] using brain homogenates prepared from terminally ill scrapie strain 139A-infected mice. All animals were monitored thrice weekly for development of clinical symptoms in the course of scrapie disease progression.

### Tissue collection

Mice were sacrificed at 6, 9, 12, and 18 months of age by cervical dislocation. Brains were removed and divided sagitally. One hemibrain was snap frozen in liquid nitrogen and stored at -80°C before further Western blot, while the other hemibrain was fixed in paraformaldehyde (4% for 24 h and 2% for additional 2–14 days) at 4°C followed by dehydration and embedding in paraffin.

### Western blot analysis

For Western blot detection of Abeta, brains were homogenized in sterile phosphate buffered saline (PBS) containing protease inhibitor (Complete ULTRA Tablets; Roche) and phosphatase inhibitor (phosphatase inhibitor cocktail 2; Sigma-Aldrich). 10% (w/v) homogenates were sonicated three times for 5 s and centrifuged at 3000 x g for 5 min at 4°C as previously described. The supernatants were collected and stored at -80°C for further analysis. For the extraction of insoluble subcellular fractions, an amount of supernatant was ultra-centrifuged at 100.000 x g for 1 h at 4°C. The pellet was resuspended in 1% PBS/ sodium dodecyl sulfate (SDS) solution, sonicated three times for 5 s and ultra-centrifuged at 100000 x g for 1 h at 4°C. The supernatant was removed and the pellet was resuspended in 88% formic acid solution (Merck). The sample was sonicated for 5 min at 4°C followed by shaking at 23°C and 600 rpm for 4 h and then stored frozen at -80°C until use.

Protein concentration was quantified by BCA protein assay (Thermo Fisher Scientific). Samples were run on 10% or 12.5% SDS-PAGE gels except for Abeta detection, where the samples were loaded on modified SDS-PAGE gels as described previously [19]. Abeta protein loads were detected with anti-Abeta 6E10 antibody (SIG-39300; Covance), microglia with anti- ionized calcium-binding adapter molecule 1(Iba1) antibody (PA5-18039; Thermo Fisher Scientific), astrocytes with anti-glial fibrillary acidic protein (GFAP) antibody (Z0334; Dako), complement component C3 with anti-C3 antibody (ICN55444; Thermo Fisher Scientific), and beta-actin (loading control) with anti-beta-actin antibody (A5441; Sigma-Aldrich). Misfolded, disease-specific PrP^Sc^ protein loads were detected using monoclonal mouse 4H11 antibody [20], kindly provided by H. Schätzl (Department of Comparative Biology and Experimental Medicine, Faculty of Veterinary Medicine, University of Calgary, Calgary, Canada).

Anti-goat AP-conjugated (705-055-147; Dianova) or anti-mouse AP-conjugated (D0486; Dako) secondary antibodies were used for Abeta protein and microglia or astrocytes and complement C3 or beta-actin detection, respectively. Signal was detected by chemiluminescence using the CDP-Star substrate (Life Technologies). All blots were analyzed using Quantity One software (Bio-Rad).

### Histology and Immunohistochemistry

Serial sagittal sections (6 μm) were cut from paraffin-embedded brains to evaluate the plaque burden and the amount of activated astrocytes and microglia. Plaque staining with Congo red (Sigma-Aldrich) was performed as described previously [21, 22]. Abeta plaques were also detected immunohistochemically using anti-Abeta 4G8 antibody (SIG-39220; BioLegend) after pretreating tissues in 70% formic acid solution for 10 min. Activated astrocytes were detected using GFAP antiserum (Z0334; Dako) and microglia were stained with the Iba-1 antibody (016–20001; Wako Chemicals). Secondary antibodies were either anti-mouse IgG (RPN1001; GE Healthcare) or anti-rabbit IgG (RPN1004; GE Healthcare). Staining was visualized using streptavidin-horseradish peroxidase conjugate (RPN1231; GE Healthcare), followed by 3,3'-Diaminobenzidine (DAB) as substrate.

For the quantification of amyloid plaque loads as well as numbers of activated astrocytes and microglia activation, 6 μm brain slices were examined under a Zeiss Axioskop-40 microscope and images were acquired at 100X magnification with a Zeiss Axiocam high resolution digital color camera. For each animal, three sections in the premotor cortex (M2) and in the hippocampal area [23] were analyzed using ImageJ software (National Institutes of Health, USA). Pictures were converted to 8-bit black and white and a fixed intensity threshold was applied defining the specific Congo red or DAB staining.

### RNA extraction and real-time PCR

Total RNA was isolated from brains using TRIzol reagent (Life Technologies), and from cell cultures using the InviTrap Spin Cell RNA Mini Kit (Stratec) according to the manufacturers’ protocols. Total RNA was reverse transcribed using the First-Strand cDNA synthesis kit (Qiagen). Real-time PCRs were performed on ABI 7500 (Applied Biosystems) and MX3000P (Stratagene) real-time PCR system cyclers using gene-specific TaqMan gene expression assays (Tables 1 and 2) and gene specific primers (Tables 3 and 4) in combination with a SYBR green dye-based gene expression detection kit (Life Technologies). Data analysis was performed by applying the delta-delta *Ct* method [24]. Two endogenous housekeeping genes, glyceraldehyde-3-phosphate dehydrogenase (GAPDH) and beta-actin, were quantified in parallel to compensate for variations in amounts of input RNA and efficiencies of reverse transcription and results were normalized to these averaged endogenous housekeeping gene values.

**Table 1:** Murine TaqMan assays.

Gene Symbol	Assay ID	Amplicon (bp)	Reference sequence
Actb	Mm00607939_s1	115	NM_007393.3
Iba-1	Mm00479862_g1	82	NM_019467.2
C3	Mm00437838_m1	70	NM_009778.2
Ccl3	Mm00441258_m1	78	NM_011337.2
Cd11b	Mm01271259_g1	73	NM_008401.2
Cxcl2	Mm00436450_m1	67	NM_009140.2
Gapdh	Mm99999915_g1	107	NM_008084.2
Gfap	Mm01253033_m1	75	NM_010277.3
Il-6	Mm00446190_m1	78	NM_031168.1

Actb, beta actin; Iba-1, ionized calcium-binding adapter molecule 1; C3, complement component C3; Ccl3, chemokine (C-C motif) ligand 3; Cd11b, cluster of differentiation molecule 11b; Cxcl2, chemokine (C-X-C motif) ligand 2, Gapdh, glyceraldehyde-3-phosphate dehydrogenase; Gfap, glial fibrillary acidic protein; Il-6, interleukin 6

**Table 2:** Human TaqMan assays.

Gene Symbol	Assay ID	Amplicon (bp)	Reference sequence
ACTB	Hs03023943_g1	122	NM_001101.3
CCL3	Hs00234142_m1	53	NM_002983.2
CP	Hs00236810_m1	85	NM_000096.3
CXCL2	Hs00601975_m1	100	NM_002089.3
CXCL9	Hs00171065_m1	60	NM_002416.1
GAPDH	Hs99999905_m1	122	NM_002046.3
IL-6	Hs00985639_m1	66	NM_000600.3

ACTB, beta-actin; CCL3, chemokine (C-C motif) ligand 3; CP, ceruloplasmin; CXCL2, chemokine (C-X-C motif) ligand 2; CXCL9, chemokine (C-X-C motif) ligand; GAPDH, glyceraldehyde-3-phosphate dehydrogenase; IL-6, interleukin 6

**Table 3:** Mouse qRT-PCR Primer.

Gene Symbol	Forward Primer 5'—-3'	Reverse Primer 5'—-3'	Amplicon (bp)	Reference sequence
Ace1	TGGCCCAGCGGCAGCAGTA	CCCTCCCAGGCAAACAACAAC	172	NM_207624.5
Actb	ACTCTTCCAGCCTTCCTTC	ATCTCCTTCTGCATCCTGTC	171	NM_007393.3
ApoE	GAGGACACTATGACGGAAGTAAA	TCTGTGCTCTGGCCCAGCATG	199	NM_009696.3
Cp	GACAACACCACTGATTTTCAACGG	TCTCCAGGACTTGGCTCATTGG	95	NM_007752.3
CtsB	AGCCATTTCTGACCGAACCT	TTGTCCAGAAGCTCCATGCT	146	NM_007798.3
Cxcl9	AAAACTGAAATCATTGCTACACTG	CTCTTTTGCTTTTTCTTTTGGCTG	125	NM_008599.4
Ece1	ACACCGACAAATGTCTGCTCAA	CCGTGTCACTCACACAAAACTT	168	NM_199307.2
Gapdh	CCATGTTTGTGATGGGTGTGAACCA	ACCAGTGGATGCAGGGATGATGTTC	251	NM_008084.2
Ide	CCGGCCATCCAGAGAATAGAA	ACGGTATTCCCGTTTGTCTTCA	69	NM_031156.2
Lrp1	CGCCATGGGGAAGGTGTTCTT	CCCTTCGTAGTCTACCACCTCG	204	NM_008512.2
Mme	CTTGTCTTGCTCCTGACTATCAT	TCAAAATTACTGTATCGGGAACT	224	NM_008604.3
Ager	TAGAATGGAAACTGAACACAGGA	GTTAGTTGCCCGACACCGG	161	NM_007425.3
Saa3	CCAGAGAGGCTGTTCAGAAGTTCAC	TCGGAAGTGGTTGGGGTCTTTG	107	NM_011315.3
Tnfaip6	CAGCTAGAGGCAGCCAGAAAA	TCCATAATCGATGATACCCGTT	135	NM_009398.2

Ace1, angiotensin I-converting enzyme 1; Actb, beta-actin; ApoE, apolipoprotein E; Cp, ceruloplasmin; CtsB, cathepsin; Cxcl9, chemokine (C-X-C motif) ligand 9; Ece1, endothelin-converting enzyme 1; Gapdh, glyceraldehyde-3-phosphate dehydrogenase; Ide, insulin-degrading enzyme; Lrp1, low density lipoprotein receptor-related protein 1; Mme, membrane metallo-endopeptidase; Ager, advanced glycosylation end product-specific receptor; Saa3, serum amyloid A 3; Tnfaip6, tumor necrosis factor alpha induced protein 6

**Table 4:** Human qRT-PCR primer.

Gene Symbol	Forward Primer 5'—-3'	Reverse Primer 5'—-3'	Amplicon (bp)	Reference sequence
C3	TACTACACGCTGATCGGTGC	GCTTTTTACCACCAGCGAGC	102	NM_000064.2
ACTB	CTGGAACGGTGAAGGTGACA	AAGGGACTTCCTGTAACAATGCA	140	NM_001101.3
GAPDH	TGGTATCGTGGAAGGACTCATGAC	ATGCCAGTGAGCTTCCCGTTCAGC	189	NM_002046.4
TNFAIP6	TGCTGCTGGATGGATGGCTA	CACTCCTTTGCGTGTGGGTT	159	NM_007115.3

ACTB, beta-actin; GAPDH, glyceraldehyde-3-phosphate dehydrogenase; Tnfaip6, tumor necrosis factor alpha induced protein 6

### Cell culture and isolation of primary mouse mixed glia cells

Mixed glia cultures were established using previously described methods [25, 26] with few modifications. In brief, brains from 1- to 3-d-old neonatal C/EBPD^(-/-)^ and WT mice were removed and cerebral cortices, cerebelli and brain stems were collected in Hank's Balanced Salt Solution (HBSS) with Mg^2+^ and Ca^2+^ (Life Technologies). Tissues were digested in 0.1% trypsin-EDTA (Biochrom) in Mg^2+^/Ca^2+^-free HBSS for 12 min at 37°C. The digestion was stopped by adding culture medium (DMEM with high Glucose (41966–029; Life Technologies) supplemented with 10% FCS (Biochrom), 100 IU/ml penicillin and 100 mg/ml streptomycin sulfate (Biochrom), and 0.1% 50 mM 2-Mercaptoethanol (Merck). Tissues were then digested in DNase I (Roche) solution and gently dissociated by pipetting. After centrifugation at 300 x g for 5 min the supernatant was removed and the pellet resuspended and passed through a 70 μm nylon cell strainer (BD Biosciences). The single-cell suspension was seeded in poly-L-lysine (0.1 mg/ml) (Sigma-Aldrich) precoated 75 cm^2^ tissue culture flasks (BD Biosciences) in culture medium. Cells were incubated at 37°C in a 95% humidified 5% CO_2_ atmosphere. One day after isolating the cells, the medium was completely replaced and cultures were kept for another 10–14 days.

### Culture and transfections of U-373 MG glioma cells

A human C/EBPD expression vector (pcMV6 human C/EBPD; SC110852, Origene) was transfected into the human glioblastoma-astrocytoma cell line U-373 MG (kind gift from N. Holtkamp, Department of Neuropathology, Charité—Universitätsmedizin Berlin, Berlin, Germany). 10^5^ cells/well were transfected using Lipofectamine LTX & Plus Reagent (Life Technologies) according to the manufacturer’s protocol and incubated for 48 h.

### Statistical analysis

Results are expressed as means ± SEM. All data were analyzed for statistical significance by two-tailed unpaired t-test using Prism 5 software (GraphPad Software Inc.). Values of p<0.05 were considered to be statistically significant.

## Results

To assess the role of C/EBPD in chronic neurodegenerative diseases APP/PS1 double transgenic mice were compared to APP/PS1 x C/EBPD^(-/-)^ mice. In addition, the intracerebral scrapie infection of wild-type C/EBPD^(+/+)^ mice was studied in comparison to similarly infected C/EBPD^(-/-)^ animals.

Overall, C/EBPD^(-/-)^ mice displayed neither in the AD nor in the prion model overt clinical differences compared to the respective C/EBPD^(+/+)^ controls. E.g. scrapie-infected C/EBPD^(-/-)^ mice developed typical clinical symptoms (weight loss, progressive ataxia, poor coat condition), just like their wild-type counter-parts. Moreover, the survival times in both groups were virtually identical (S1 Table). Similarly, in the AD model body weights and nesting activities were indistinguishable between both groups of mice (S1 Fig).

**Fig 1:**
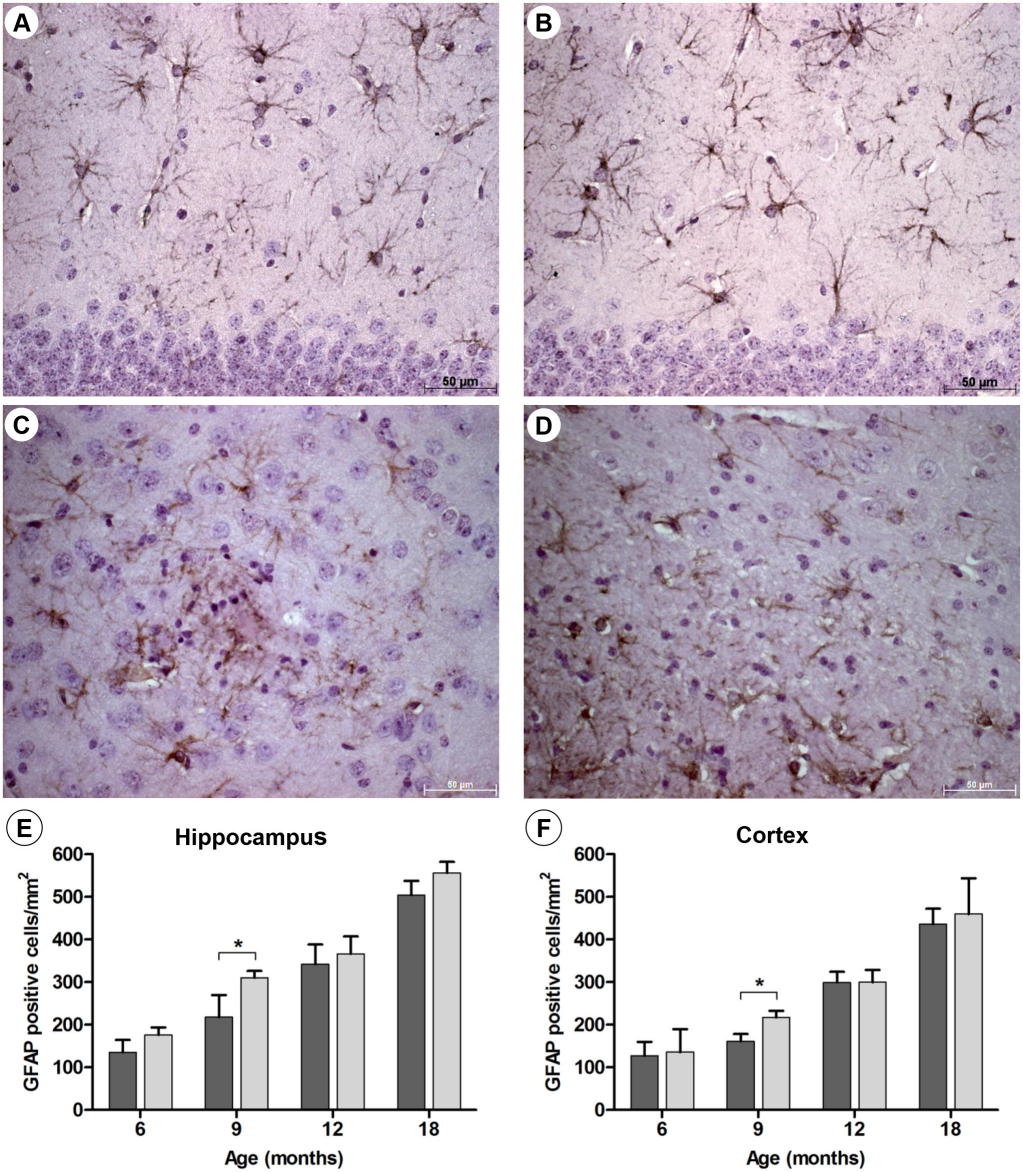
Astrocytosis in APP/PS1 and APP/PS1 x C/EBPD^(-/-)^ mice. Representative images showing the detection of GFAP-positive astrocytes in hippocampi (**A** and **B**) and cortices (**C** and **D**) at 9 months of age in APP/PS1 mice (**A** and **C**) and APP/PS1 x C/EBPD^(-/-)^ mice (**B** and **D**). Quantification of GFAP-positive astrocyte numbers in hippocampi (**E**) and cortices (**F**) over time in APP/PS1 mice (n = 3; dark grey bars) and APP/PS1 x C/EBPD^(-/-)^ mice (n = 3; light grey bars).

**Fig 2:**
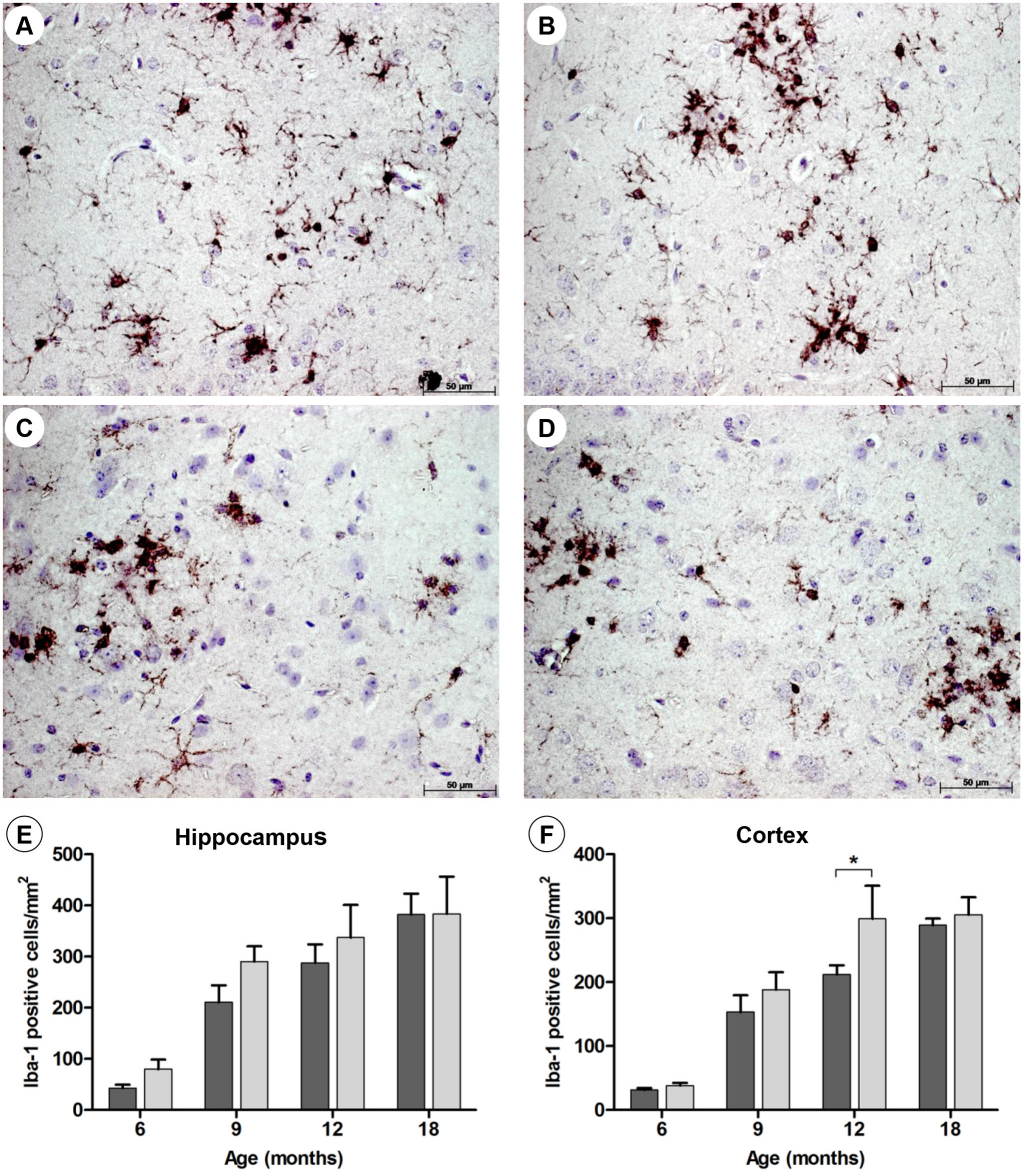
Microgliosis in APP/PS1 and APP/PS1 x C/EBPD^(-/-)^ mice. Representative images showing the detection of Iba-1-positive microglia in hippocampi (**A** and **B**) and cortices (**C** and **D**) at 12 months of age in APP/PS1 mice (**A** and **C**) and APP/PS1 x C/EBPD^(-/-)^ mice. Quantification of Iba-1-positive cell numbers in hippocampi (**E**) and cortices (**F**) over time in APP/PS1 mice (n = 3; dark grey bars) and APP/PS1 x C/EBPD^(-/-)^ mice (n = 3; light grey bars).

Next, the extent of the astro- and microgliosis in the AD and prion models was studied in C/EBPD^(-/-)^ and C/EBPD^(+/+)^ mice. Astroglial GFAP and microglial Iba-1 expression was determined by immunohistochemistry (Figs 1 and 2), by Western blot analysis as well as by quantitative RT-PCR (S2 and S3 Figs). All animals showed typical age- and disease-related increases of GFAP and Iba-1 expression levels over time, which were however hardly influenced by the presence or absence of C/EBPD. Moreover, the mRNA expression of the microglial activation marker CD11b was found to be identical in brains of APP/PS1 x C/EBPD^(-/-)^ and APP/PS1 x C/EBPD^(+/+)^ animals (S10 Fig). Likewise, glia activation in scrapie-infected C/EBPD^(-/-)^ and wild type mice appeared to be indistinguishable (S12 Fig).

**Fig 3:**
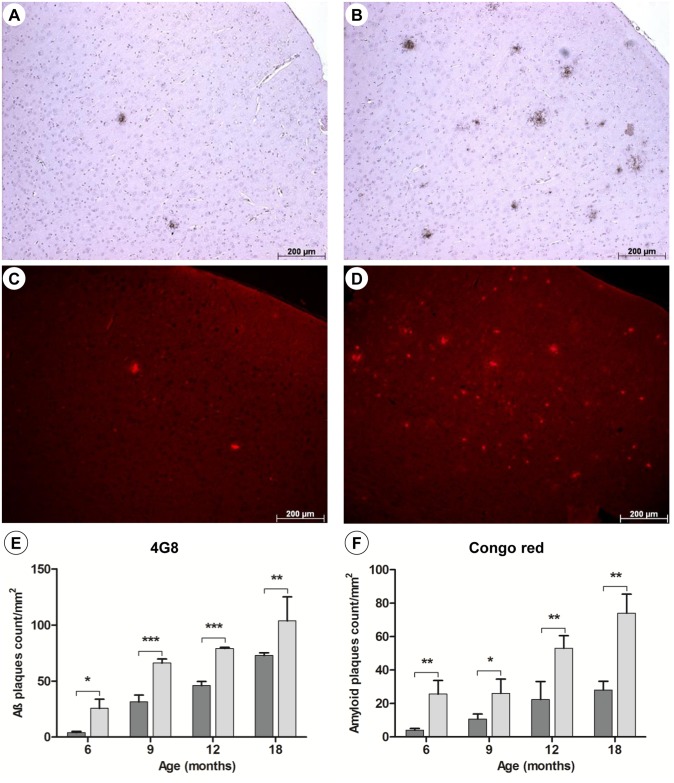
Amyloid plaque loads in premotor cortices (M2). Representative images of amyloid plaques detected at 6 months of age using the 4G8 antibody (**A** and **B**) or Congo red staining (**C** and **D**) in APP/PS1 mice (**A** and **C**) and APP/PS1 x C/EBPD^(-/-)^ mice (**B** and **D**). Quantification of cortical plaque loads over time using 4G8 antibody staining (**E**) and Congo red staining (**F**) in APP/PS1 mice (n = 3; dark grey bars) and APP/PS1 x C/EBPD^(-/-)^ mice (n = 3; light grey bars).

**Fig 4:**
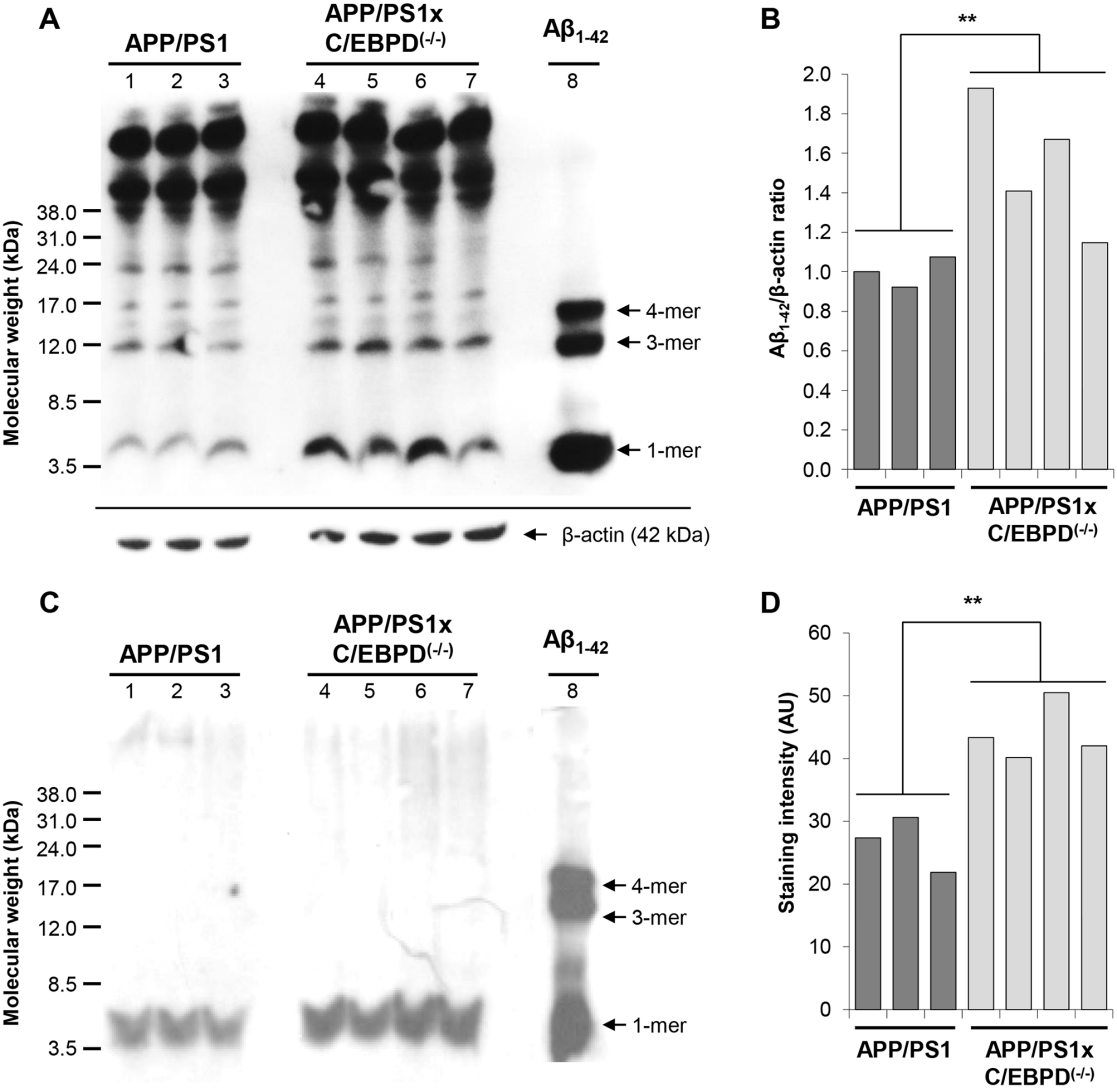
Western blot detection of Abeta in brain extracts from APP/PS1 and APP/PS1 x C/EBPD^(-/-)^ mice. Abeta protein loads in PBS-soluble (**A**) and formic acid-soluble (**C**) brain extracts detected using the 6E10 antibody at 9 months of age. Synthetic Abeta1-42 peptide served as additional size marker. (**B** and **D**) Densitometric quantification of monomeric Abeta band intensities from (**A**) and (**C**), respectively (**p<0.01). Of note, the overloaded high molecular weight material in the upper third of the gel in (**A**) consists of amyloid precursor protein and unspecific bands and is not relevant for the comparison of Abeta amounts.

While in the prion model, PrP^Sc^-levels in C/EBPD^(-/-)^ mice were similar to C/EBPD^(+/+)^ mice (S11 Fig), immunohistochemical analysis of Abeta-immunoreactive plaques using the 6E10 antibody revealed an increased plaque burden in C/EBPD^(-/-)^ compared to C/EBPD^(+/+)^ mice (Fig 3). The higher plaque load in C/EBPD-deficient mice was evident at all ages analyzed and was most strongly pronounced in the cortex region. Staining of true amyloid plaques with Congo red confirmed these observations (Fig 3). Amyloid plaque numbers increased over time in cortices of mice from both groups but were significantly higher at all ages in C/EBPD-deficient animals (*p<0.05; **p<0.01; at 6**, 9*, 12** and 18** months) (Fig 3). In addition, the amounts of total Abeta in brain homogenates from C/EBPD^(-/-)^ and C/EBPD^(+/+)^ mice were assessed by Western blotting. In agreement with the plaque load data PBS- as well as formic acid-extractable Abeta levels were significantly higher (p<0.05; p<0.01) in the C/EBPD^(-/-)^ group of mice (Fig 4).

Importantly, the higher levels of Abeta deposition in C/EBPD-deficient mice were not attributable to an increase in APP expression (S4 Fig). In addition, mRNA expression levels of genes known to contribute to clearance and degradation of Abeta were determined. No differences between the C/EBPD^(-/-)^ and C/EBPD^(+/+)^ animals were detectable concerning the expression of neprilysin (membrane metallo-endopeptidase; MME), apolipoprotein E (Apoe), low density lipoprotein receptor-related protein 1 (Lrp1), cathepsin B (Ctsb), endothelin-converting enzyme 1 (Ece1), angiotensin I-converting enzyme (Ace), insulin-degrading enzyme (Ide), and advanced glycosylation end product-specific receptor (Ager; formerly termed RAGE). Hence, the increased Abeta deposition in C/EBPD^(-/-)^ mice was not caused by alterations in expression levels of genes involved in turnover or transport of Abeta (S5 Fig).

**Fig 5:**
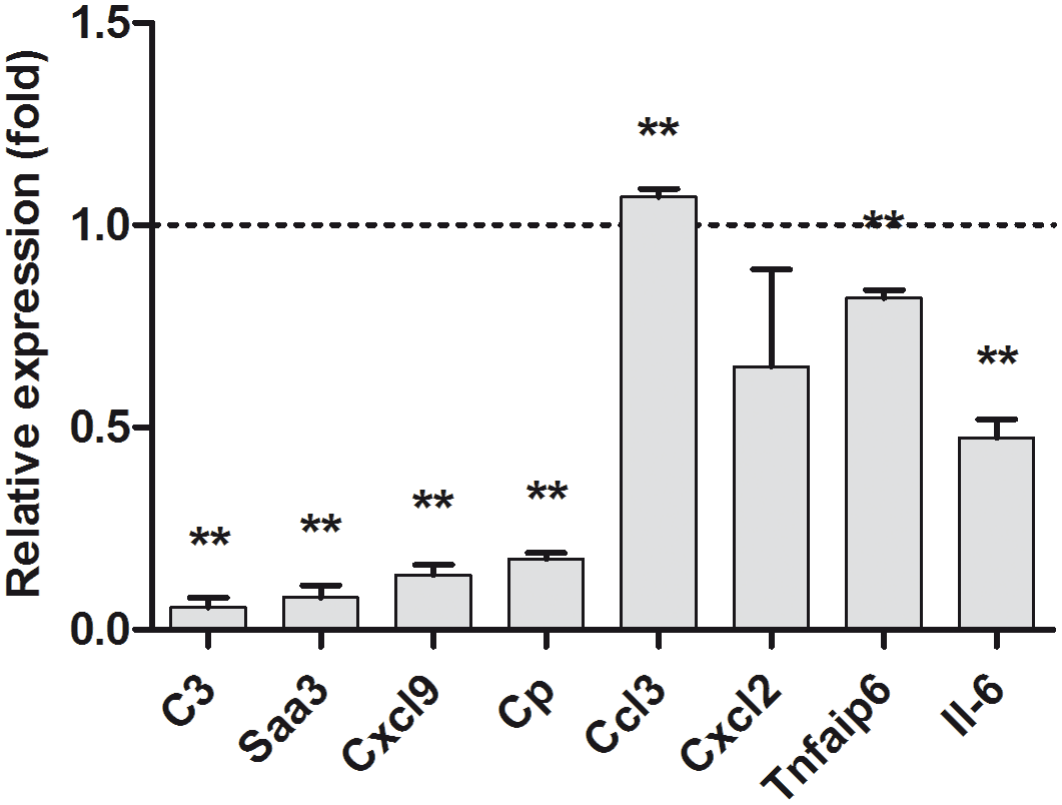
Expression of putative C/EBPD target genes in mixed glia. Gene expression levels in C/EBPD^(-/-)^ mixed glial cultures relative to wild type C/EBPD^(++)^ mixed glial cultures (**p<0.01). The broken line indicates the expression level in wild type cultures.

To gain more insight into C/EBPD functions in the CNS, we focused next on the expression of candidate genes previously suggested to be regulated by the transcription factor C/EBPD in the periphery [2, 3, 8, 10, 12, 27–29]. Moreover, these genes were selected because of their potential participation in innate immune responses triggered by amyloid deposition in the brain. Mixed glia cultures were established from C/EBPD^(-/-)^ and C/EBPD^(+/+)^ donor mice and mRNA expression levels for a total of 8 genes were determined (Fig 5). Expression of C3 was most strikingly affected by the absence of C/EBPD (20-fold decreased expression, p<0.01), while for others (serum amyloid A 3 (Saa3), chemokine (C-X-C motif) ligand 9 (Cxcl9), ceruloplasmin (Cp)) 5.56–12.5-fold decreases (p<0.01) were observed. In addition, expression of a further group of C/EBPD candidate target genes was more or less identical (decrease less than 2-fold) in the presence or absence of C/EBPD (chemokine (C-C motif) ligand 3 (Ccl3), Cxcl2, tumor necrosis factor alpha induced protein 6 (Tnfaip6), interleukin 6 (Il-6)). Given that C3 mRNA levels were most clearly dependent on the presence of C/EBPD we looked in addition for C3 protein expression by immunoblotting. In agreement with the quantitative RT-PCR data, C3 and C3b protein was only detectable in C/EBPD^(+/+)^ mixed glia lysates but was virtually absent in C/EBPD^(-/-)^ cells (S6 Fig).

Expression analysis of the same genes in brains from APP/PS1 and APP/PS1 x C/EBPD^(-/-)^ mice confirmed that C3 mRNA levels depend on C/EBPD expression (p<0.01), but changes for all other genes were lower than 1.5-fold and less or not significant (S7 Fig). Likewise, C3 transcript levels showed also the most pronounced differences in comparisons of mRNA levels in scrapie-infected C/EBPD^(+/+)^ and C/EBPD^(-/-)^ mouse brains (S8 Fig).

Further support for the idea that C3 transcription in the CNS is indeed driven by C/EBPD was obtained by ectopic expression of C/EBPD in the human glioblastoma cell line U-373 MG, which expresses the astrocyte marker GFAP [30]. Upon transfection of a C/EBPD expression vector transcript levels of the putative target genes C3, CXCL9, CCL3, and TNFAIP6 showed very robust increases (> 5-fold) over empty control vector transfections, while others (CP and IL-6) were less inducible (<5-fold) or even unresponsive (CXCL2) (S9 Fig).

## Discussion

In the periphery the transcription factor C/EBPD acts as part of a regulatory circuit to drive and amplify innate immune responses triggered by bacterial infections [2, 31, 32].

Chronic neurodegenerative illnesses like AD and prion diseases have a significant inflammatory component in part resulting from the activation of elements of the innate immunity [33–35]. However, a possible involvement of C/EBPD in neuroinflammation or, more generally, in disease progression was to our knowledge as yet largely underinvestigated. We characterized here mice deficient for C/EBPD to learn more about a possible role of this transcription factor in AD and in prion diseases.

In terms of AD-like pathology the single and most profound alteration in APP/PS1 x C/EBPD^(-/-)^ mice compared to APP/PS1 x C/EBPD^(+/+)^ mice was the increased plaque load at all stages of the disease, which was also reflected by elevated amounts of Abeta in corresponding brain extracts. Moreover, the significantly higher plaque load in the C/EBPD-deficient mice was evident in immunohistochemical antibody stainings as well as in Congo red-based detection of true amyloid plaques. In contrast, the accumulation of misfolded proteinase K-resistant prion protein PrP^Sc^ in scrapie-infected C/EBPD^(-/-)^ mice compared to similarly infected wild-type animals was unchanged. Therefore it seems that C/EBPD does not influence protein misfolding events in general but may contribute in a more specific manner to the deposition of Abeta. Interestingly, a previous study mentioned no difference in cortical plaque numbers between APP/PS1 x C/EBPD^(-/-)^ and APP/PS1 x C/EBPD^(+/+)^ mice [36]. However, given that the underlying data have as yet not been published it is for the time being not possible to provide explanations for this potentially conflicting result.

The higher plaque load in APP/PS1 x C/EBPD^(-/-)^ mice observed in our study was not associated with changes concerning clinical symptoms and/or behavioral deficits. However, more detailed studies would be needed to address possible learning and memory impairments in these animals. In scrapie-infected mice ablation of C/EBPD had no effect on disease duration, development of clinical symptoms, and survival times.

Theoretically, one would expect in C/EBPD-deficient mice a more pronounced gliosis in response to the higher amyloid plaque burden. If however C/EBPD functions in part as driver of glial activation these inflammatory glial responses would be attenuated in the absence of C/EBPD. In terms of overall cell numbers and morphology the absence of C/EBPD had no influence on the extent of the astro- and microgliosis, which was found to be essentially identical in C/EBPD^(+/+)^ and C/EBPD^(-/-)^ mice (Figs 1 and 2). Moreover, mRNA and protein expression levels of astroglial GFAP and microglial Iba-1 were virtually identical in C/EBPD-deficient mice compared to the controls (S2 and S3 Figs).

Of note, our results concerning the gliosis do not rule out a local reduction of glial activation in close proximity to amyloid plaques in APP/PS1 x C/EBPD^(-/-)^ mice [37, 38]. These findings may indicate that glial migration is impaired in these animals. In support of this idea we show here that C/EBPD promotes expression of the cytokines CCL3 and CXCL9 (S9 Fig). However, our analyses of astro- and microglia activation by immunohistochemistry, Western blotting, and quantitative RT-PCR clearly argue against the previously indicated possibility of a more general attenuation of glial responses in APP/PS1 x C/EBPD^(-/-)^ mice [37, 38].

Expression studies in cell cultures and in vivo revealed that C/EBPD may indeed play a role in the regulation of specific glial activities. Overexpression of C/EBPD in U-373 MG cells was previously shown to induce acute phase pentraxin-3 (Ptx-3) transcription as well as the expression of its interaction partner Tnfaip6 [10]. Ptx-3 was suggested to inhibit phagocytosis of damaged neurons by macrophages in vitro [10]. Therefore, C/EBPD may play a role in modulating inflammatory responses triggered by tissue degeneration [32, 37]. In our hands, expression of C3, Saa3, Cxcl9, Cp, and Il-6 was significantly reduced in the absence of C/EBPD in mixed glia cultures. Vice versa, transfection of the human glioblastoma cell line U-373 MG with a C/EBPD expression vector led to a significant upregulation of C3, CXCL9, CP, CCL3, TNFAIP6, and IL-6 mRNA levels. C/EBPD may therefore play a role in regulating the expression of these genes in the CNS.

The example of Tnfaip6, which was found to be unaffected by C/EBPD in mixed glia cultures but appeared to be highly responsive to C/EBPD in U-373 MG cells, illustrates that C/EBPD functions can be cell type dependent. In addition, regulation of gene expression through C/EBPD apparently depends on the applied type of stimulation. E.g. in LPS-stimulated mixed glia cultures, expression of typically C/EBPD-regulated genes like IL-6 appear to be driven by other transcription factors than C/EBPD [9]. CCAAT/enhancer-binding protein beta (C/EBPB), which also forms heterodimers with C/EBPD, is a potential candidate in this regard [12, 39].

Among the genes studied here we found that only C3 and Saa3 mRNA levels were affected by C/EBPD in mouse brain tissue. Transcript levels of C3 and Saa3 were significantly lower in brain tissue from APP/PS1 x C/EBPD^(-/-)^ mice and scrapie-infected C/EBPD^(-/-)^ mice compared to the respective controls. Of note, in the CNS both genes are typically overexpressed by activated astrocytes [12, 40], which correlates well with the described astrocytic overexpression of C/EBPD in AD and AD model mice [7, 10]. Hence, the evidence presented here indicates that C/EBPD is a transcriptional regulator of the acute phase response genes C3 and Saa3 in vivo. The observed induction of glial C3 overexpression by the pro-inflammatory cytokines IL-1 and TNFalpha may therefore well be attributable to C/EBPD [41]. Anyhow, given that C3 and Saa3 expression was still detectable in brain tissue of C/EBPD^(-/-)^ mice, it is clear that other transcription factors, e.g. C/EBPB [39], can at least to some extent compensate for the absence of C/EBPD.

A number of reports have linked complement component C3 to AD pathogenesis [42, 43]. C3 expression is upregulated in AD affected brain tissue [44, 45] and is important for the phagocytosis and clearance of fibrillar Abeta [46, 47]. Specifically, ablation of C3 led to a significant reduction of Abeta uptake in glial cell cultures [46, 47]. Accordingly, in murine AD models inhibition of C3 activation as well as C3 a deficiency leads to increased plaque formation [42, 43]. Thus, the reduced C3 expression levels observed in APP/PS1 x C/EBPD^(-/-)^ mice may well explain the higher plaque load in these animals. Reduced levels of C3 mRNA were also observed in brain tissue from scrapie-infected C/EBPD^(-/-)^ mice. In peripheral prion infections a deficiency for C3 delays splenic prion accumulation and prolongs survival times [48–50]. However, while C3 promotes peripheral prion spread its role in intracerebral prion infections is less clear [51]. We show here that a reduction of C3 levels in mice intracerebrally infected with scrapie strain 139A has no influence on survival or deposition of misfolded prion protein PrP^Sc^. Given that C3 expression is clearly upregulated in prion-infected brain tissue [13, 52] larger studies using mice with a genetic ablation of this complement component seem warranted to ultimately clarify this issue.

Taken together, we show here that a C/EBPD-deficiency leads to increased Abeta plaque burden in AD model mice. Furthermore, as shown in vivo and in vitro, C/EBPD is an important driver of the expression of the acute phase response genes C3 and Saa3 in the amyloid-affected CNS. In future work an elucidation of the pathways, which regulate C/EBPD in response to the Abeta burden, may well help to deepen our understanding of pathomechanisms in fatal chronic neurodegenerative diseases like AD.

## Supporting Information

S1 Fig Development of body weights and nest building activity in the AD model. (**A**) Body weights and (**B**) nest building activity scores of APP/PS1 mice (n = 3; dark grey circles) and APP/PS1 x C/EBPD^(-/-)^ mice (n = 3; light grey triangles) were monitored from 3 to 12 months of age.
(TIF)

S2 Fig Astrocytic GFAP expression in mouse brains (AD model). (**A**) Western blot detection of GFAP protein from 18-month-old APP/PS1 (n = 3) and APP/PS1 x C/EBPD^(-/-)^ mice (n = 4). (**B**) Densitometric quantification of the blot shown in (**A**) for APP/PS1 mice (dark grey bars) and APP/PS1 x C/EBPD^(-/-)^ mice (light grey bars). (**C**) Comparison of GFAP levels detected by Western blotting in APP/PS1 x C/EBPD^(-/-)^ mice relative to APP/PS1 mice at different time points. (**D**) Determination of GFAP mRNA levels in APP/PS1 x C/EBPD^(-/-)^ mice relative to APP/PS1 mice (n = 3).
(TIF)

S3 Fig Microglial Iba-1 expression in mouse brains (AD model). (**A**) Western blot detection of Iba-1 protein in brain homogenates from 18-month-old APP/PS1 and APP/PS1 x C/EBPD^(-/-)^ mice. (**B**) Densitometric quantification of Iba-1 levels from (**A**) for APP/PS1 mice (n = 3; dark grey bars) and APP/PS1 x C/EBPD^(-/-)^ mice (n = 4; light grey bars). (**C**) Comparison of Iba-1 levels detected by Western blotting in APP/PS1 x C/EBPD^(-/-)^ mice relative to APP/PS1 mice at different time points. (**D**) Determination of Iba-1 mRNA levels in APP/PS1 x C/EBPD^(-/-)^ mice relative to APP/PS1 mice (n = 3).
(TIF)

S4 Fig APP expression in APP/PS1 and APP/PS1 x C/EBPD^(-/-)^ mice. (**A**) Western blot using brain homogenates from 9 month-old mice. (**B**) Densitometric quantification of APP band intensities from (**A**) in APP/PS1 mice (n = 2; dark grey bars) and APP/PS1 x C/EBPD^(-/-)^ mice (n = 3; light grey bars).
(TIF)

S5 Fig Brain expression levels of genes involved in Abeta transport and turnover. Relative expression levels in APP/PS1 x C/EBPD^(-/-)^ mice compared to APP/PS1 mice at 18 months of age (n = 3).
(TIF)

S6 Fig Expression of C3 and C3b protein in mixed glia obtained from C/EBPD^(+/+)^ and C/EBPD^(-/-)^ mice. Western blot detection of full length C3 proteins (186.5 kDa) and cleaved C3b fragment (102 kDa) in mixed glia cell lysates. Of note, C3 and C3b protein is hardly detectable in C/EBPD^(-/-)^ cell lysates.
(TIF)

S7 Fig Brain expression of putative C/EBPD target genes (AD model). Expression levels in APP/PS1 x C/EBPD^(-/-)^ mice relative to APP/PS1 mice at 18 months of age (n = 3).
(TIF)

S8 Fig Brain expression of putative C/EBPD target genes (Prion model). Expression levels in scrapie-infected C/EBPD^(-/-)^ mice (n = 3) relative to similarly infected wild type mice at the terminal stage of the disease.
(TIF)

S9 Fig Expression of putative C/EBPD target genes in U-373MG cells transfected with a C/EBPD expression vector. Expression levels shown are relative to empty vector control transfections. Of note, expression of the human SAA3 gene was not included in this experiment because it is considered to be a non-functional, non-transcribed pseudogene (Kluve-Beckerman B, Drumm ML, Benson MD. Nonexpression of the human serum amyloid A three (SAA3) gene. DNA Cell Biol. 1991;10(9):651–61. PubMed PMID: 1755958).
(TIF)

S10 Fig CD11b mRNA levels in brain homogenates. Determination of CD11b mRNA levels in APP/PS1 x C/EBPD^(-/-)^ mice (n = 3) relative to APP/PS1 mice (n = 3).
(TIF)

S11 Fig Western blot detection of proteinase K resistant PrP^Sc^ in brain extracts from scrapie-infected wild type C/EBPD^(+/+)^ and C/EBPD^(-/-)^ mice. (**A**) Western blot from scrapie-infected C/EBPD^(+/+)^ (n = 3) and C/EBPD^(-/-)^ mice (n = 3) at the terminal stage of the disease. (**B**) Densitometric quantification of staining intensities.
(TIF)

S12 Fig Astrocytosis and microgliosis in scrapie-infected wild type C/EBPD^(+/+)^ and C/EBPD^(-/-)^ mice. Representative images showing GFAP-positive astrocytes (**A** and **B**) and Iba-1-positive microglia (**C** and **D**) at 125 dpi in hippocampi of scrapie-infected wild type C/EBPD^(+/+)^ (**A** and **C**) and C/EBPD^(-/-)^ mice (**B** and **D**). Quantification of GFAP-positive astrocytes (**E**) and Iba-1-positive microglia (**F**) at 125 dpi in wild type C/EBPD(+/+) mice (n = 4; dark grey bars) and C/EBPD(-/-) mice (n = 4; light grey bars).
(TIF)

S1 Table Survival times (Prion model). Survival times of scrapie-infected wild type C/EBPD^(+/+)^ (n = 9) and C/EBPD^(-/-)^ mice (n = 9).
(TIF)
