# Supplementary figures and images for: Ablation of CCAAT/Enhancer-Binding Protein Delta (C/EBPD): Increased Plaque Burden in a Murine Alzheimer’s Disease Model
Source: PLoS One. 2015 Jul 31;10(7):e0134228. doi: 10.1371/journal.pone.0134228 (PMC4521790; doi:10.1371/journal.pone.0134228)

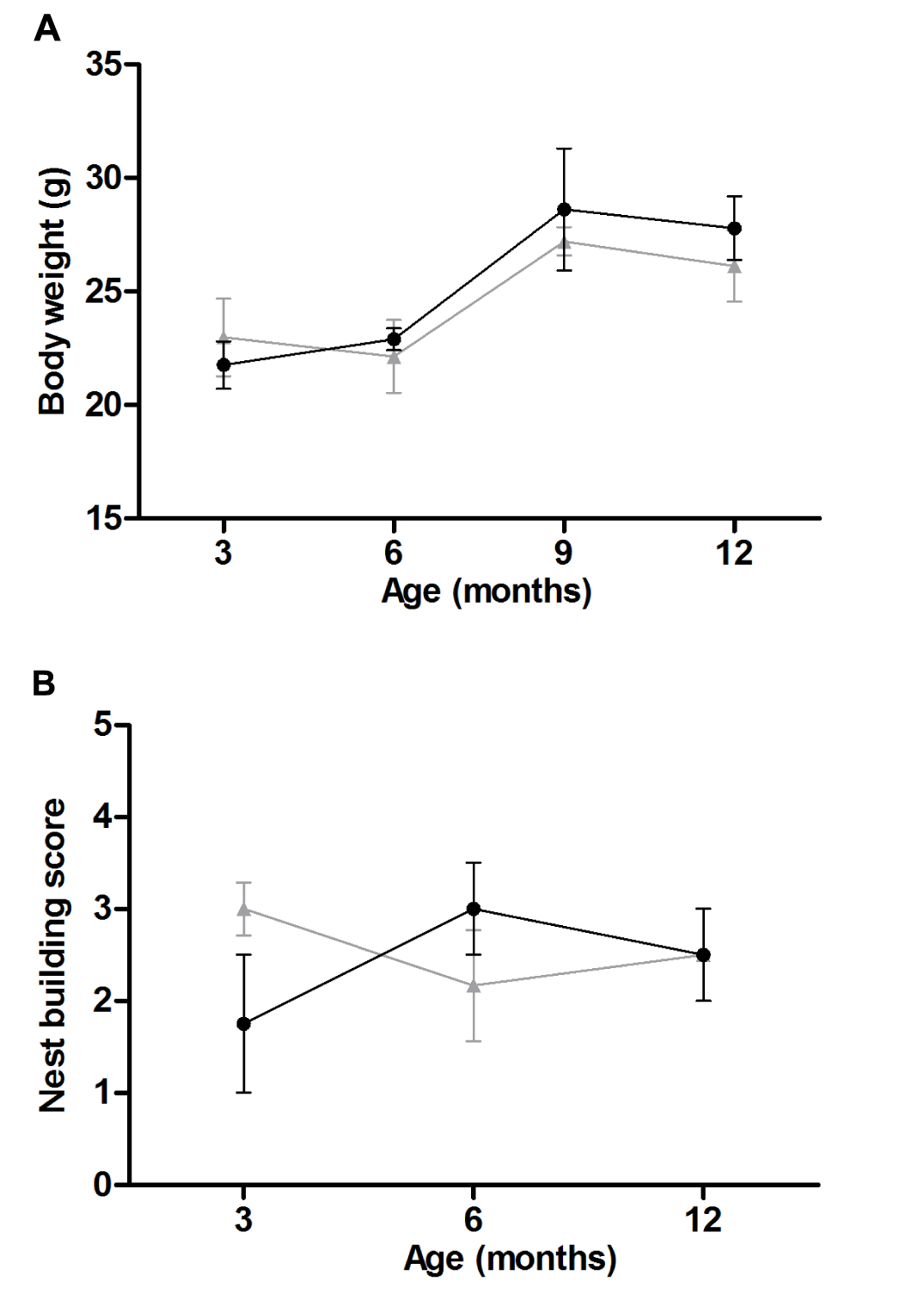

Supplement: S1 Fig — (A) Body weights and (B) nest building activity scores of APP/PS1 mice (n = 3; dark grey circles) and APP/PS1 x C/EBPD(-/-) mice (n = 3; light grey triangles) were monitored from 3 to 12 months of age. (TIF) [file pone.0134228.s001.tif]

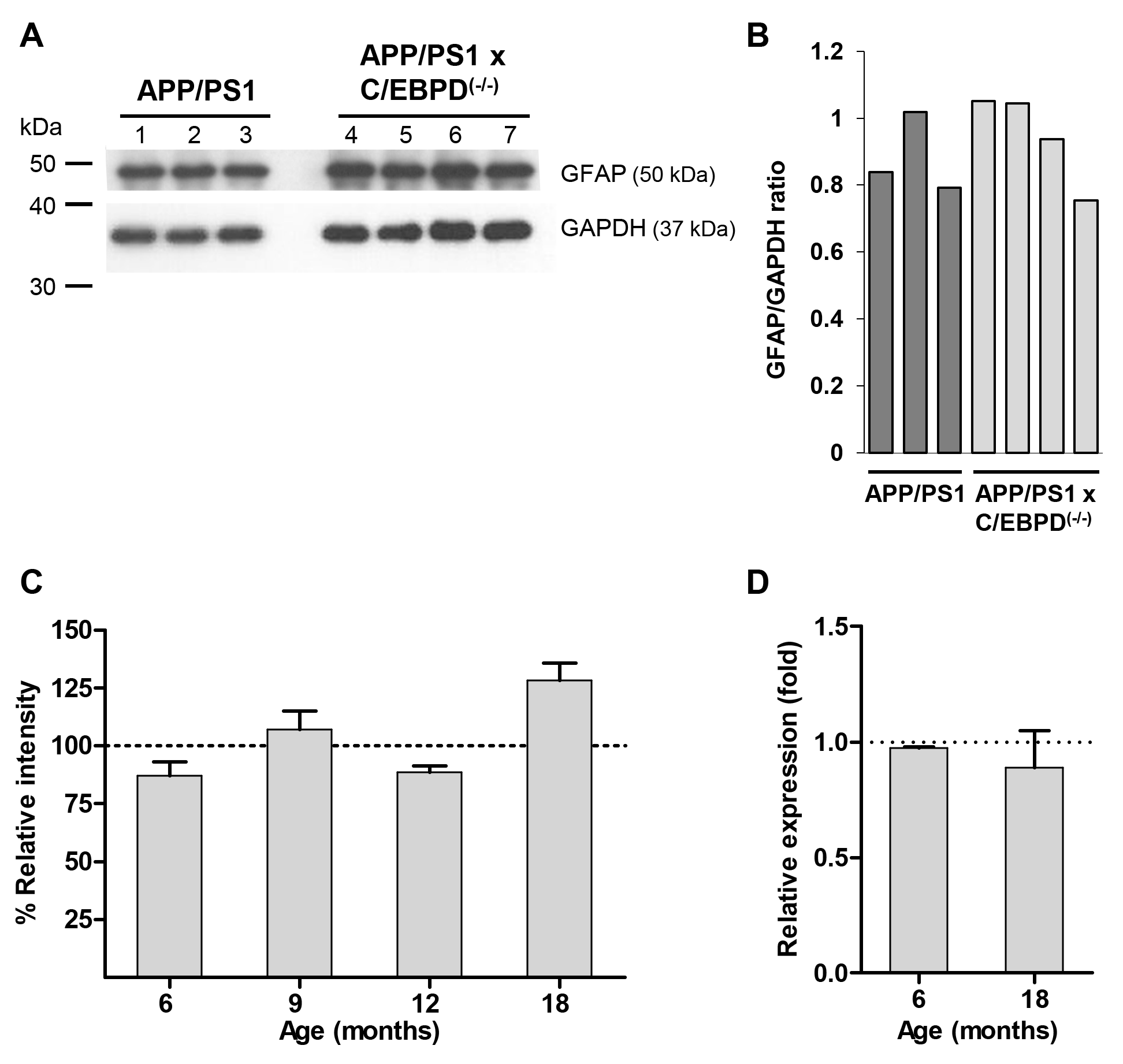

Supplement: S2 Fig — (A) Western blot detection of GFAP protein from 18-month-old APP/PS1 (n = 3) and APP/PS1 x C/EBPD(-/-) mice (n = 4). (B) Densitometric quantification of the blot shown in (A) for APP/PS1 mice (dark grey bars) and APP/PS1 x C/EBPD(-/-) mice (light grey bars). (C) Comparison of GFAP levels detected by Western blotting in APP/PS1 x C/EBPD(-/-) mice relative to APP/PS1 mice at different time points. (D) Determination of GFAP mRNA levels in APP/PS1 x C/EBPD(-/-) mice relative to APP/PS1 mice (n = 3). (TIF) [file pone.0134228.s002.tif]

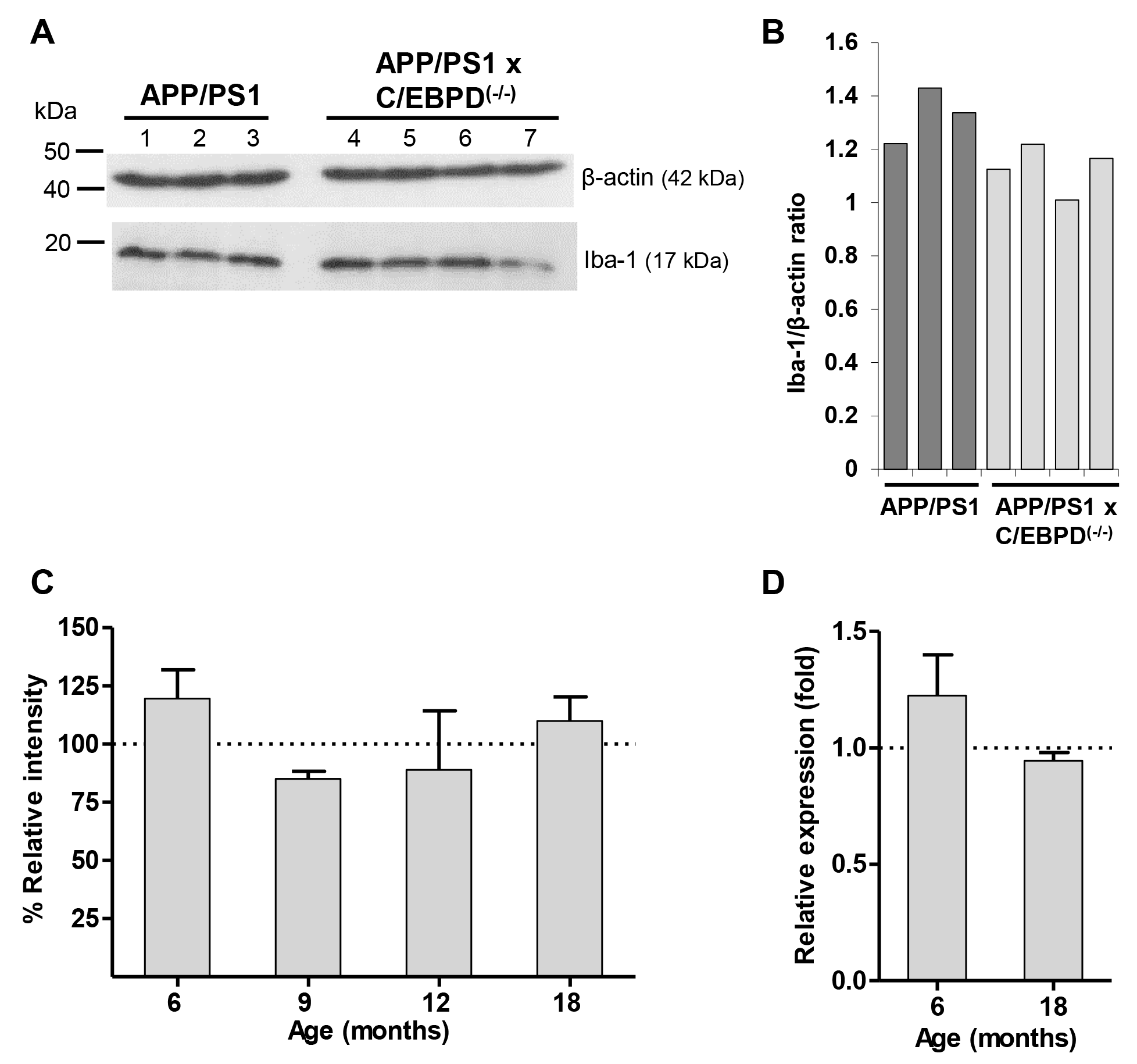

Supplement: S3 Fig — (A) Western blot detection of Iba-1 protein in brain homogenates from 18-month-old APP/PS1 and APP/PS1 x C/EBPD(-/-) mice. (B) Densitometric quantification of Iba-1 levels from (A) for APP/PS1 mice (n = 3; dark grey bars) and APP/PS1 x C/EBPD(-/-) mice (n = 4; light grey bars). (C) Comparison of Iba-1 levels detected by Western blotting in APP/PS1 x C/EBPD(-/-) mice relative to APP/PS1 mice at different time points. (D) Determination of Iba-1 mRNA levels in APP/PS1 x C/EBPD(-/-) mice relative to APP/PS1 mice (n = 3). (TIF) [file pone.0134228.s003.tif]

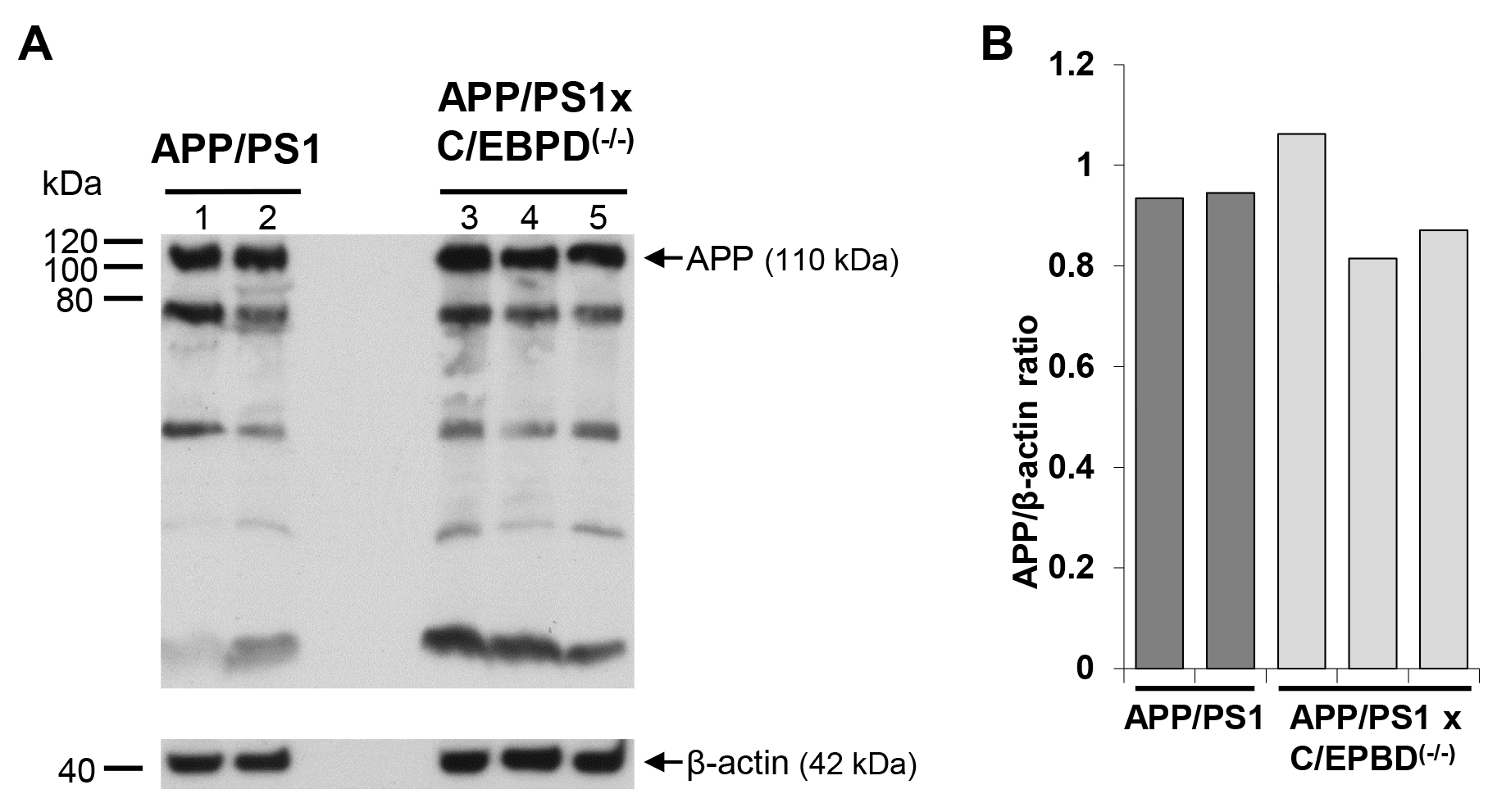

Supplement: S4 Fig — (A) Western blot using brain homogenates from 9 month-old mice. (B) Densitometric quantification of APP band intensities from (A) in APP/PS1 mice (n = 2; dark grey bars) and APP/PS1 x C/EBPD(-/-) mice (n = 3; light grey bars). (TIF) [file pone.0134228.s004.tif]

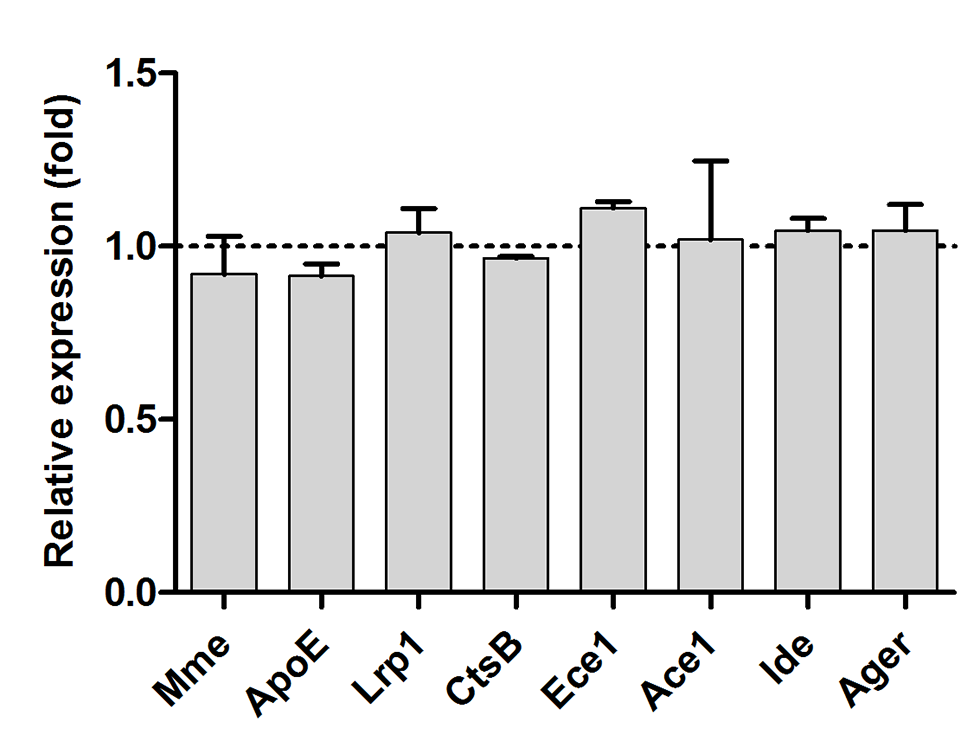

Supplement: S5 Fig — Relative expression levels in APP/PS1 x C/EBPD(-/-) mice compared to APP/PS1 mice at 18 months of age (n = 3). (TIF) [file pone.0134228.s005.tif]

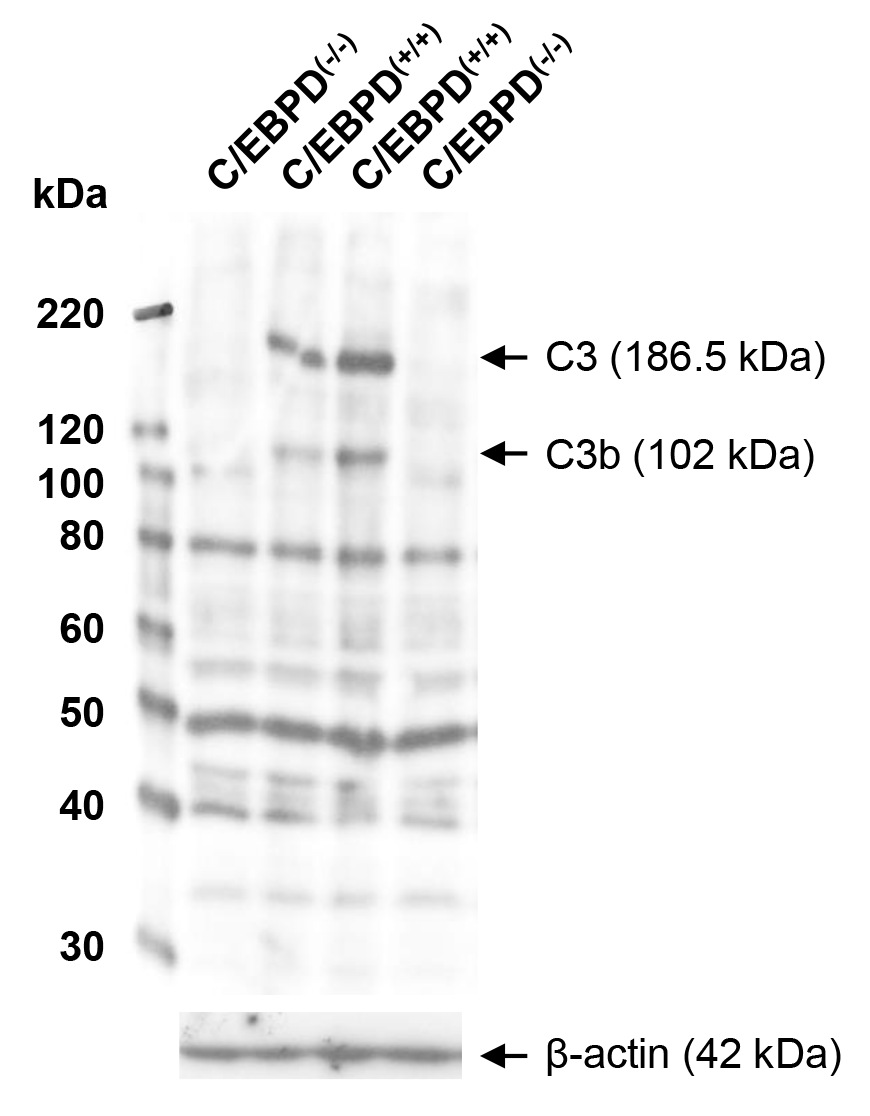

Supplement: S6 Fig — Western blot detection of full length C3 proteins (186.5 kDa) and cleaved C3b fragment (102 kDa) in mixed glia cell lysates. Of note, C3 and C3b protein is hardly detectable in C/EBPD(-/-) cell lysates. (TIF) [file pone.0134228.s006.tif]

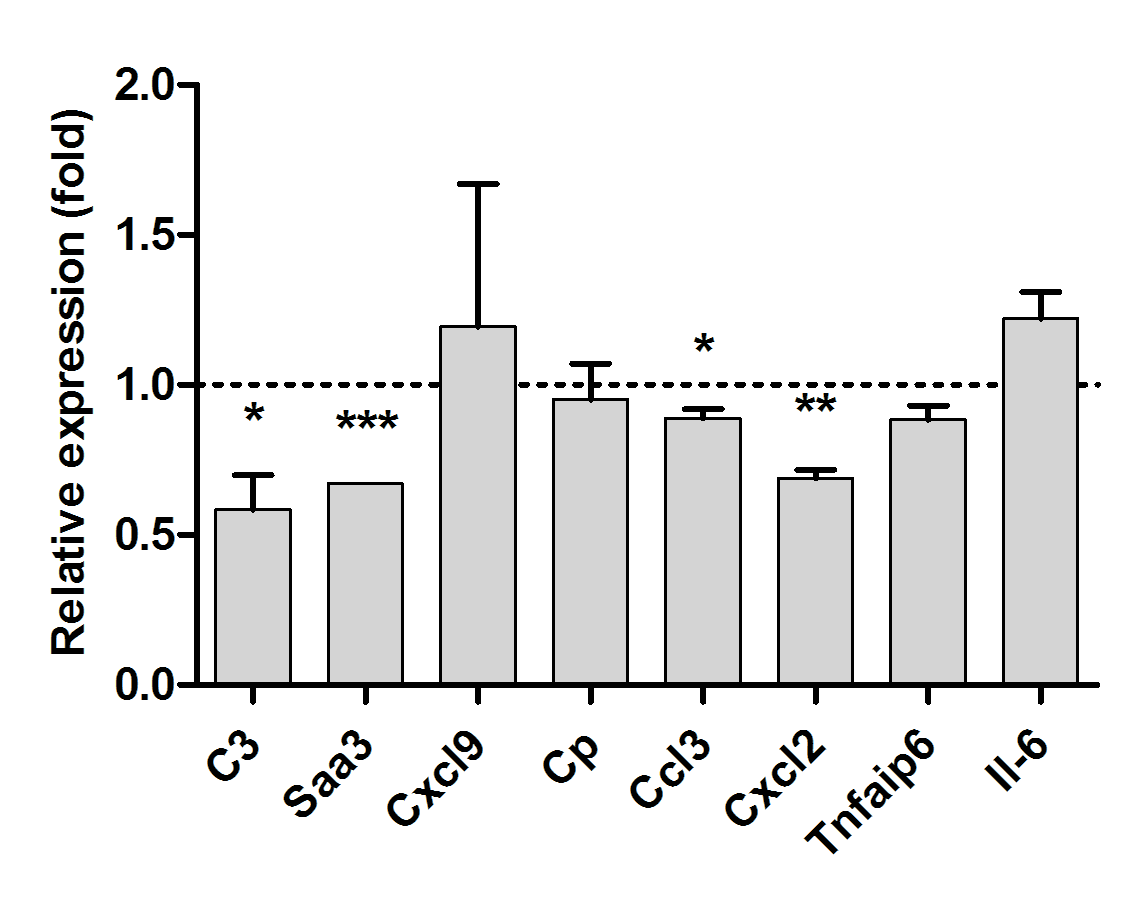

Supplement: S7 Fig — Expression levels in APP/PS1 x C/EBPD(-/-) mice relative to APP/PS1 mice at 18 months of age (n = 3). (TIF) [file pone.0134228.s007.tif]

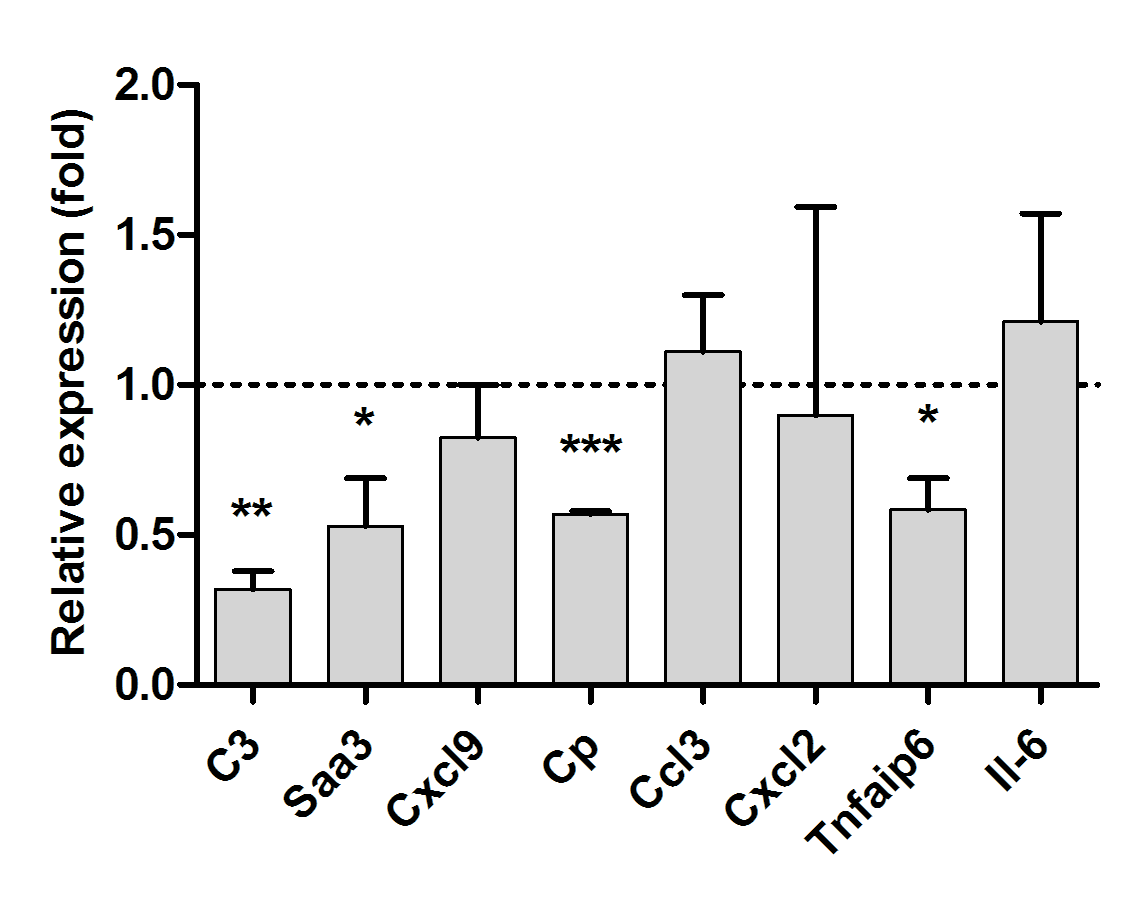

Supplement: S8 Fig — Expression levels in scrapie-infected C/EBPD(-/-) mice (n = 3) relative to similarly infected wild type mice at the terminal stage of the disease. (TIF) [file pone.0134228.s008.tif]

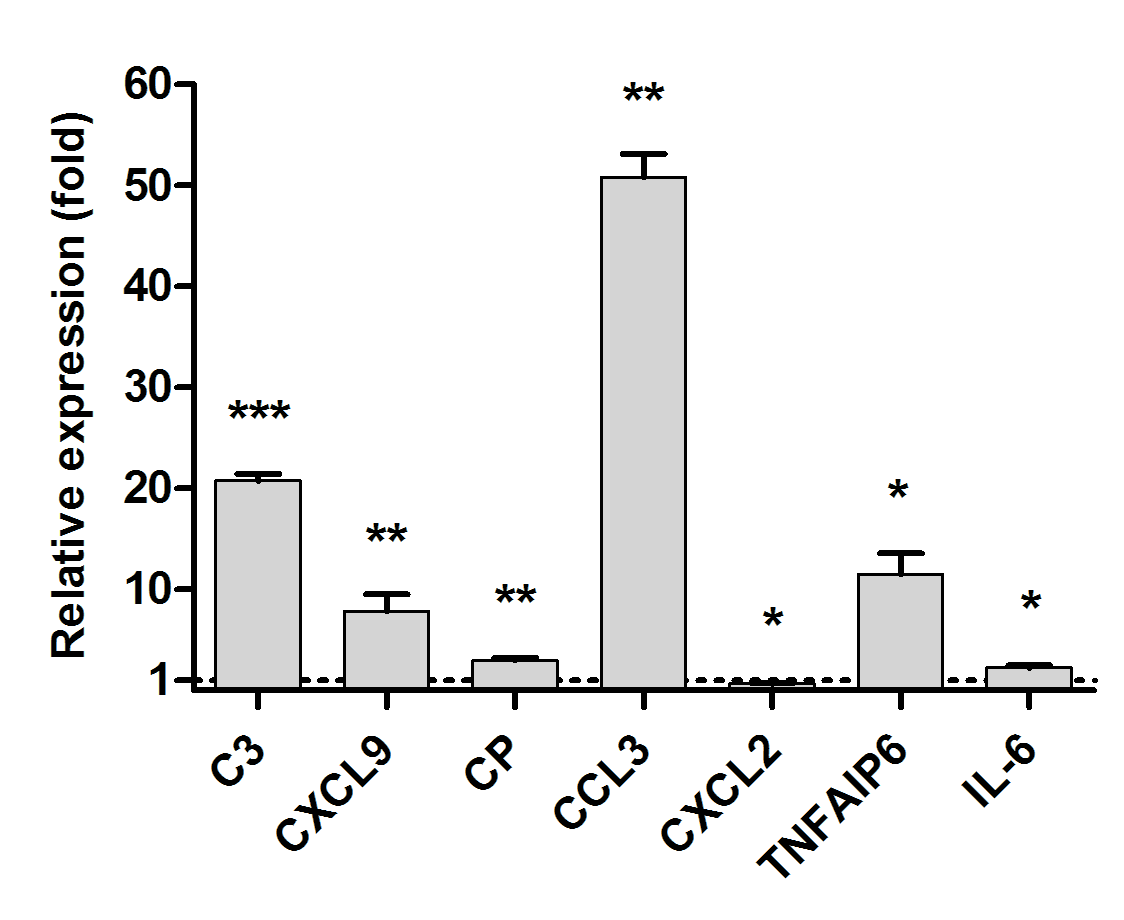

Supplement: S9 Fig — Expression levels shown are relative to empty vector control transfections. Of note, expression of the human SAA3 gene was not included in this experiment because it is considered to be a non-functional, non-transcribed pseudogene (Kluve-Beckerman B, Drumm ML, Benson MD. Nonexpression of the human serum amyloid A three (SAA3) gene. DNA Cell Biol. 1991;10(9):651–61. PubMed PMID: 1755958). (TIF) [file pone.0134228.s009.tif]

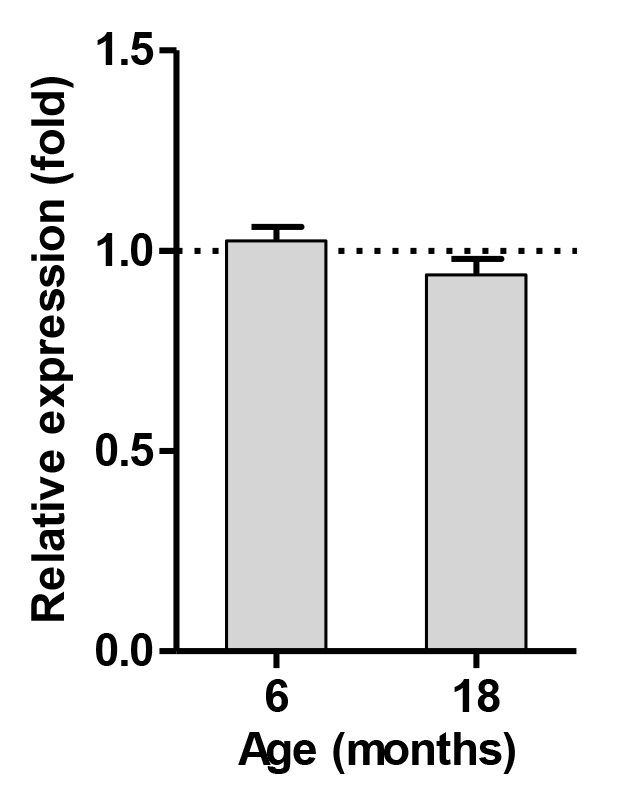

Supplement: S10 Fig — Determination of CD11b mRNA levels in APP/PS1 x C/EBPD(-/-) mice (n = 3) relative to APP/PS1 mice (n = 3). (TIF) [file pone.0134228.s010.tif]

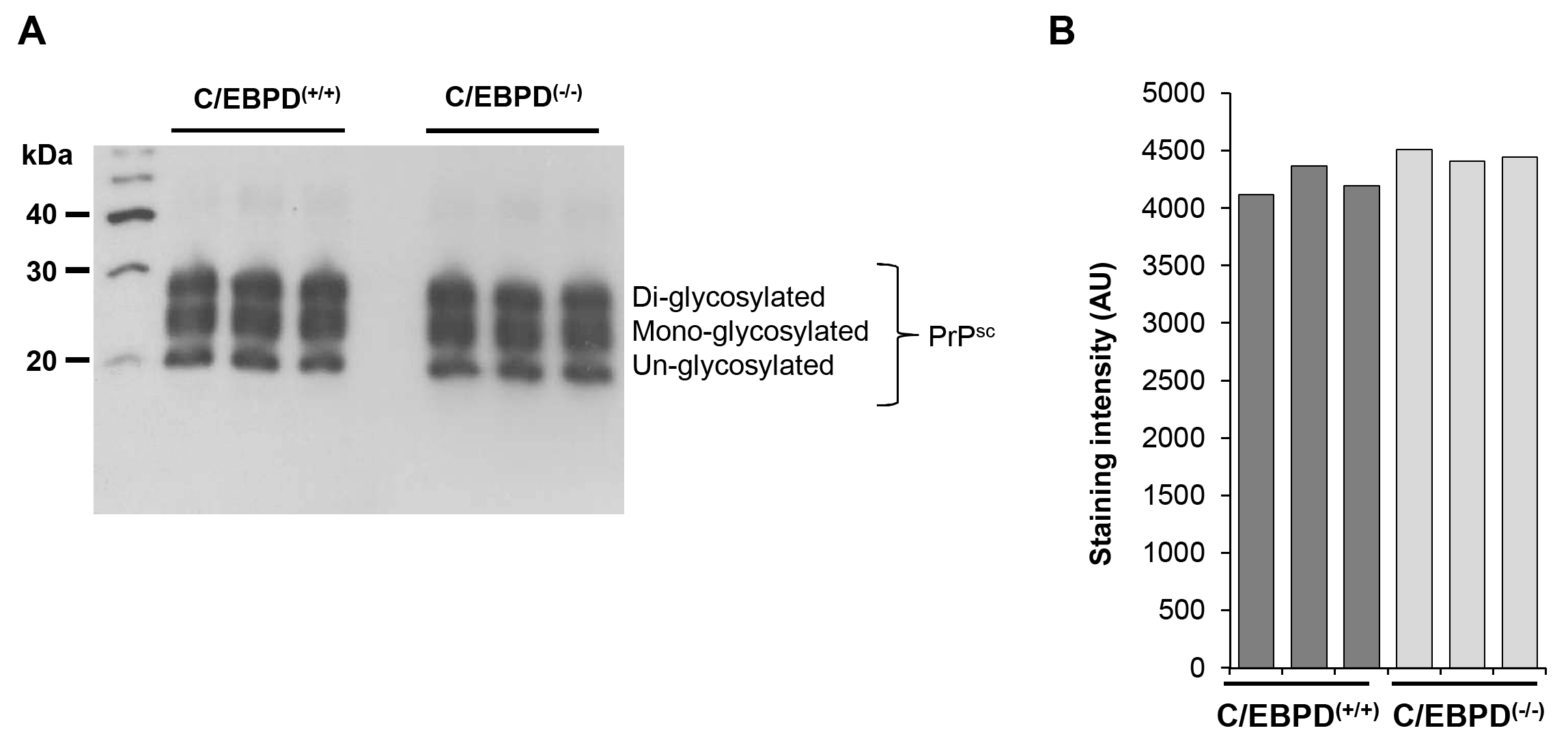

Supplement: S11 Fig — (A) Western blot from scrapie-infected C/EBPD(+/+) (n = 3) and C/EBPD(-/-) mice (n = 3) at the terminal stage of the disease. (B) Densitometric quantification of staining intensities. (TIF) [file pone.0134228.s011.tif]

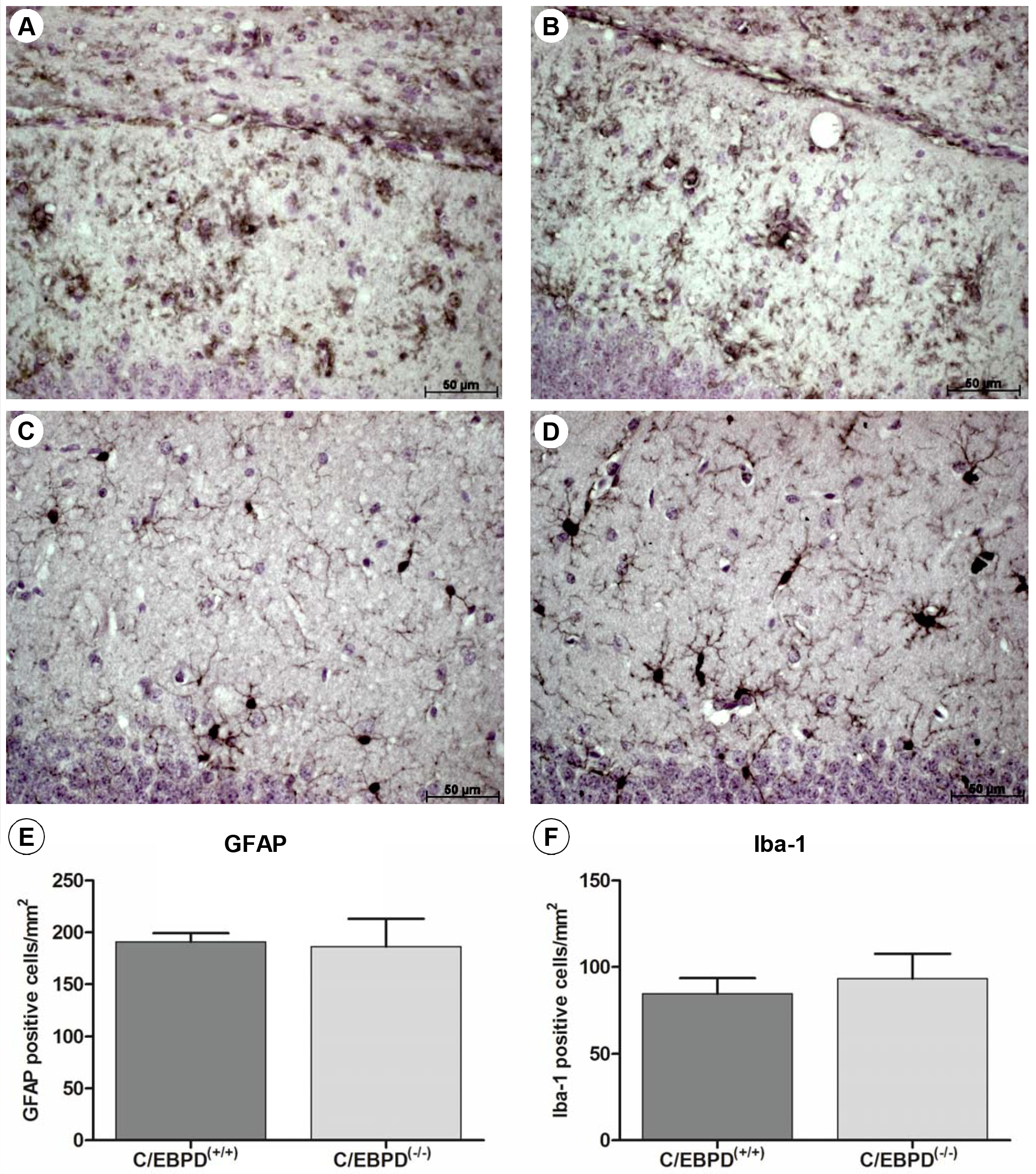

Supplement: S12 Fig — Representative images showing GFAP-positive astrocytes (A and B) and Iba-1-positive microglia (C and D) at 125 dpi in hippocampi of scrapie-infected wild type C/EBPD(+/+) (A and C) and C/EBPD(-/-) mice (B and D). Quantification of GFAP-positive astrocytes (E) and Iba-1-positive microglia (F) at 125 dpi in wild type C/EBPD(+/+) mice (n = 4; dark grey bars) and C/EBPD(-/-) mice (n = 4; light grey bars). (TIF) [file pone.0134228.s012.tif]

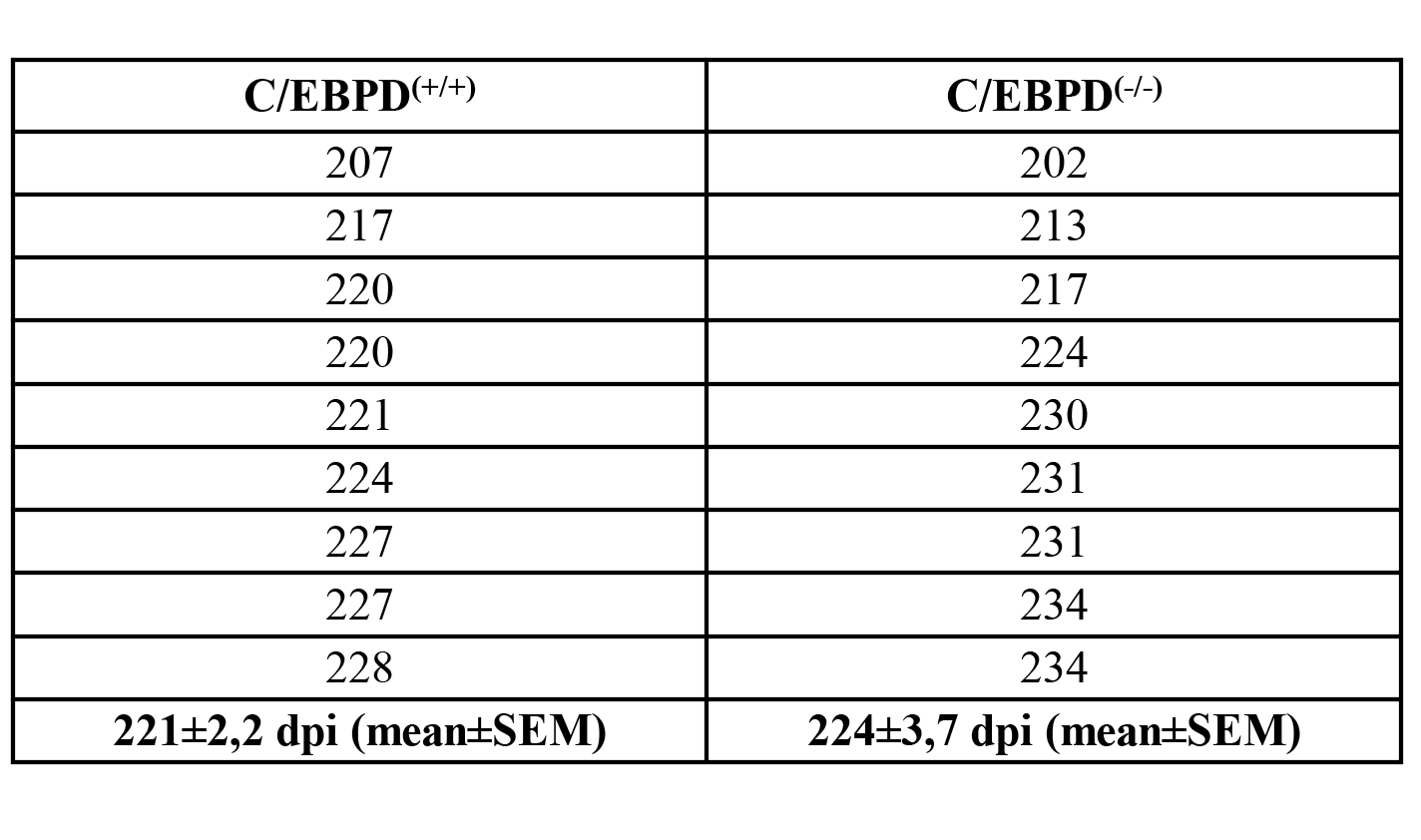

Supplement: S1 Table — Survival times of scrapie-infected wild type C/EBPD(+/+) (n = 9) and C/EBPD(-/-) mice (n = 9). (TIF) [file pone.0134228.s013.tif]
